# Pioneering telesurgery in gynecology: the first European case of total hysterectomy

**DOI:** 10.1007/s11701-025-02638-1

**Published:** 2025-08-08

**Authors:** Enrico Pazzaglia, Pietro Pasquini, Emily Jamaer, Koen Traen, Evelyn Despierre, Alexandre Mottrie

**Affiliations:** 1 ORSI Academy, Melle, Belgium; 2 Division of Gynecology and Human Reproduction Physiopathology, IRCCS Azienda Ospedaliero-Universitaria di Bologna, Via Massarenti 13, 40138 Bologna, Italy; 3 Department of Medical and Surgical Sciences (DIMEC), University of Bologna, Via Massarenti 13, 40138 Bologna, Italy; 4 Division of Oncologic Gynecology, IRCCS Azienda Ospedaliero-Universitaria di Bologna, Via Massarenti 13, 40138 Bologna, Italy; 5 Department of Gynecology and Obstetrics, AZORG Hospital, Aalst, Belgium; 6 Department of Urology, AZORG Hospital, Aalst, Belgium

**Keywords:** Telesurgery, Gynecology, Robotic surgery, Toumai system, Remote surgery

## Abstract

This report describes the first European clinical case of robot-assisted telesurgery in gynecology using the Toumai® Endoscopic Surgery System. A 34-year-old woman with recurrent cervical glandular intraepithelial neoplasia underwent a total hysterectomy with bilateral salpingectomy. The procedure was performed remotely between two institutions in Belgium, approximately 20 km apart. The remote surgeon operated from a dedicated console, while the patient and the surgical team were on site. The operation was completed successfully without intraoperative complications or conversion. Total operative time was 74 min, with a console time of 47 min. The mean latency was 20 ms with a jitter < 10 ms, with high image quality and no connection issues. Postoperative recovery was uneventful, and the patient was discharged on day 2. At the 18-day follow-up, no complications were observed. Subjective feedback highlighted a high level of satisfaction among the patient and surgical staff, with the remote surgeon reporting a slightly reduced sense of control, mitigated by the on-site stand-by surgeon. Poor audio quality was noted as a minor issue. This first experience confirms that telesurgery in gynecology is technically feasible and safe, even using a standard network connection. Although further improvements and larger studies are needed, this experience supports telesurgery as a promising tool to expand access to expert surgical care.

## Introduction

Telemedicine has been conceptually present since the 1950 s, but only in the last two decades has it extended into surgery. Early forms included telementoring, where expert surgeons guide remote procedures [1].

The advent of robotic surgery in the 1990 s enabled high-precision remote operations, laying the foundation for telesurgery. Early 2000 s trials proved its feasibility, though limited by technical and financial barriers [2].

Two decades later, rapid improvements in telecommunications, the proliferation of robotic platforms, and global interconnectivity have revived interest in telesurgery. It is now seen as a viable strategy to bridge surgical care gaps by enabling expert surgeons to operate across geographic boundaries without requiring patient transfer. In parallel, robotic surgery has become increasingly prominent in gynecology, particularly for complex procedures requiring precision and minimally invasive access, and numerous studies have investigated the feasibility and safety of various gynecologic procedures performed using robotic platforms [3, 4].

In this evolving landscape, the Toumai® Endoscopic Surgery System (MedBot-MicroPort, Shanghai, China), certified for gynecologic use in Europe as of May 2024, was designed specifically for remote procedures. It includes latency-reduction mechanisms and control-optimization technologies for safe telesurgical performance. The system underwent preclinical testing in 2024, performing various urologic and gynecologic procedures on inanimate and live animal models.

To date, human telesurgery in gynecology is scarcely reported. This study presents the first European case of a remote Robot-Assisted Total Hysterectomy (RATH) using the Toumai® system, detailing the technical setup, surgical outcomes, and postoperative course.

## Materials and methods

The patient, a 34-year-old G4P4A0 with a BMI of 21 and a medical history significant for gastric ulcer and recurrent pneumothorax, presented with recurrent cervical glandular intraepithelial neoplasia (CGIN) following a prior conization. In this context, a total hysterectomy with bilateral salpingectomy was indicated as the definitive treatment [5]. Informed consent was obtained prior to the procedure.

The Toumai® Endoscopic Surgery System (MT-1000, MedBot–MicroPort, Shanghai, China) was used. It is a multi-arm robotic platform developed for minimally invasive and remote surgical applications. The system consists of three components on-site: a Surgeon Console, a Patient Side Platform, and a Vision Platform, while for the remote setting, a telesurgery station and an additional surgeon console are needed.

The telesurgical architecture is enabled by a proprietary, high-bandwidth, low-latency communication protocol that ensures seamless interaction between the remote surgeon’s console and the patient-side platform.

In May 2025, a telesurgery session was conducted for the gynecology and urology departments at AZORG Hospital (Aalst, Belgium). The primary surgeon operated remotely from the ORSI Academy (Melle, Belgium), while the patient, the robotic system, an additional console enabled by an automatic fallback mechanism in case of communication disruption, and the local surgical team were located on site. The two centers were approximately 20 km apart. The local surgical team was composed of a bedside assistant, a second assistant, the scrub nurse, a circulating nurse, an anesthesiologist, and an on-site stand-by surgeon, as well as technical staff from the system’s manufacturer.

The bedside assistant was responsible for creating the pneumoperitoneum, placing the trocars, docking the robot, providing intraoperative field support, managing instrument exchanges as needed, undocking the system, and performing skin closure.

An experienced robotic surgeon served as the stand-by surgeon, available on site to take over console control or assist the bedside surgeon in case of system failure.

At the end of the procedure, responses to oral questions were recorded from all participants and observers, including the remote surgeon and the on-site team. The questions focused on personal impressions regarding team communication quality, differences compared to on-site surgery, and the main limitations encountered.

These perspectives were collected as an additional qualitative layer to complement the objective technical assessment.

The technical parameters of the connection, the system’s safety measures, and the communication settings are outlined in Table 1.

**Table 1:** Technical parameters and settings of the telesurgical system

Technical parameter	
Type of connection used	Standard V-LAN
Average and peak latency (ms)	Average 20 ms, Peak < 30 ms
Jitter (latency variation over time)	< 10 ms
Throughput (data rate, Mbps)	< 24Mbps
Redundancy (backup communication channel)	Not supported
Communication protocols used (e.g., UDP, QUIC)	UDP, TCP, WebRTC, TLS1.3
Audio/video communication system between sites	MicroVision Video Conference
Data encryption protocols (e.g., TLS, DTLS)	TLS1.3
Authentication and access control (e.g., 2FA, tokens)	2FA (local login & invitation code); remote control request & confirm
Protection against cyberattacks (e.g., DDoS, MITM)	Firewall & anti-malware; Certificate-based authentication Encryption libraries;
Real-time technical monitoring system	Yes, on Control & Laparoscope video
Emergency protocols (manual switch/local takeover)	Dual-console setting; second console-surgeon on-site

This table summarizes the network and cybersecurity characteristics used during the telesurgery procedure. UDP: User Datagram Protocol; TCP: Transmission Control Protocol; WebRTC: Web Real-Time Communication; TLS: Transport Layer Security. TLS 1.3 ensures secure encryption of transmitted data. 2FA: Two-Factor Authentication. DDoS: Distributed Denial of Service; MITM: Man-In-The-Middle attacks. Manual takeover ensured by a second surgeon present on-site at a backup console

## Results

The total operative time was 74 min, of which 6 min of port placement, 13 min of docking time, 47 min of console time, and 8 min of undocking and closure time.

The procedure was completed without intraoperative complications, conversion, or the need for additional ports.

The technical infrastructure allowed for a safe and stable procedure. The communication protocols (UDP, TCP, and WebRTC) and encrypted channels (TLS 1.3) enabled real-time, high-quality video and instrument control with a mean latency of 20 ms, jitter < 10 ms, and no connection issues; those parameters were also continually monitored in real time, and the video conference system facilitated communication between sites.

The on-site team did not need to intervene beyond standard bedside assistance, but the dual-console configuration was always active and ensured immediate on-site takeover in case of need. The estimated blood loss was 40 mL and the postoperative course was uneventful with the patient being discharged on postoperative day 2. At the follow-up 18 days post-intervention, no complications were detected.

Subjective feedback from the operating room staff indicated that the procedure was largely comparable to on-site surgery, while the patient reported no greater anxiety than with standard surgical procedures. The remote surgeon noted a slightly reduced sense of control due to physical distance, partially offset by the presence of a stand-by surgeon. The lack of auditory feedback from energy devices was identified as a minor limitation. The bedside assistant reported poor audio quality affecting instruction clarity, while no specific issues were noted by the rest of the team.

## Discussion

This article presents the first Europe-to-Europe telesurgical procedure in gynecology. While several procedures have already been conducted worldwide in gynecology, detailed reports describing individual procedures and immediate outcomes remain scarce. During the first Telesurgery Consensus Conference, the Toumai® system was identified as the platform with the highest number of human telesurgical procedures performed, with over 100 cases, 7% of which were gynecological [6]. The only published gynecological outcome data come from a retrospective study of 66 cases, one of which was performed over 300 km with a mean latency of 61 ms, comparable to our experience considering the longer distance, and an operative time similar to ours (110 min vs 73 min) [7]. Compared to on-site robot-assisted hysterectomy using other platforms, our operative time was consistent with published data [8–10].

During the procedure, verbal communication was limited by suboptimal audio transmission in certain moments. To ensure clarity, we restricted spoken exchanges to essential surgical instructions only. Future improvements could involve the integration of directional microphones or enhanced audio technologies to support more continuous interaction. Telesurgery also demands a higher-than-usual level of preparation from the bedside assistants. In the worst-case scenario, they must be able to manage independently, potentially without communication with the console surgeon, all steps not directly related to console operation, and they represent the first line of response in case of emergency.

The requirement for an on-site stand-by surgeon with full expertise in robotic surgery, capable of performing the entire procedure independently, currently represents a limitation of the telesurgical approach. It not only defeats the core purpose of telesurgery, which is to enable access to specialized surgeons remotely, but also demands additional personnel resources, which may not be feasible or scalable in all clinical settings. Nevertheless, this precaution was considered necessary given the experimental nature of the procedure. As technology improves and emergency protocols become standardized, it is expected that the on-site team will only need to be trained in managing complications, rather than replicating the full skill set of the remote console surgeon. During standard on-site robotic surgery, complications are typically categorized as either hardware-related or surgical; telesurgery introduces a third category: connection issues. While surgical complications can often be managed remotely, conversion to laparoscopic or open surgery may be required and must be handled by the bedside team without the support of the console surgeon. Conversely, hardware or connection problems should be managed locally by the on-site technician, a role that is expected to become increasingly critical in the future.

Latency remains a key technical challenge in telesurgery. While < 10 ms is optimal, particularly for bleeding control, delays up to 100 ms are acceptable for motion accuracy, and < 200 ms remain safe [11]. In our case, ~ 20 ms latency resulted in a subjectively imperceptible delay between the surgeon’s input and the remote robotic response.

Although a dedicated channel connection, such as 5G or satellite links with high bandwidth, is certainly the optimal choice for performing telesurgery, our experience demonstrated that a standard V-LAN connection was highly effective, providing imperceptible latency and high image quality throughout the procedure. This suggests that, at least over short-to-medium distances, an extremely advanced internet infrastructure may not be strictly necessary, but rather only required for very long-distance procedures. This could support the broader adoption of telesurgery even in regions where telecommunication infrastructures are not yet fully developed.

Now that major technical barriers are being overcome and early procedures are emerging globally, the next challenges involve data protection, cybersecurity, and medicolegal frameworks: [12] at the moment, cross-border telesurgery lacks unified regulations, requiring case-specific agreements, and that limits the adoption of the technology on a broader scale. It will be crucial to address these aspects to make telesurgery a scalable solution capable of delivering high-quality care to all patients, regardless of location.

## Conclusions

Our experience confirms the technical feasibility and safety of robot-assisted telesurgery in gynecology using the Toumai® system, with outcomes comparable to standard procedures. Despite minor technical refinements still needed, telesurgery emerges as a viable alternative to on-site surgery. Larger studies and prospective trials are needed to validate these results, refine protocols, and define its role in gynecologic care.

## Data Availability

No datasets were generated or analyzed during the current study.
